# Green‐shifting of SWS2A opsin sensitivity and loss of function of RH2‐A opsin in flounders, genus *Verasper*

**DOI:** 10.1002/ece3.3745

**Published:** 2017-12-27

**Authors:** Satoshi Kasagi, Kanta Mizusawa, Akiyoshi Takahashi

**Affiliations:** ^1^ School of Marine Biosciences Kitasato University Kitasato Minami‐ku Sagamihara Kanagawa Japan

**Keywords:** flounder (flatfish), molecular evolution, variation, visual opsin

## Abstract

We identified visual opsin genes for three flounder species, including the spotted halibut (*Verasper variegatus*), slime flounder (*Microstomus achne*), and Japanese flounder (*Paralichthys olivaceus*). Structure and function of opsins for the three species were characterized together with those of the barfin flounder (*V. moseri*) that we previously reported. All four flounder species possessed five basic opsin genes, including *lws*,* sws1*,* sws2*,* rh1*, and *rh2*. Specific features were observed in *rh2* and *sws2*. The *rh2‐a*, one of the three subtypes of *rh2*, was absent in the genome of *V. variegatus* and pseudogenized in *V. moseri*. Moreover, *rh2‐a* mRNA was not detected in *M. achne* and *P. olivaceus*, despite the presence of a functional reading frame. Analyses of the maximum absorption spectra (λ_max_) estimated by *in vitro* reconstitution indicated that SWS2A of *M. achne* (451.9 nm) and *P. olivaceus* (465.6 nm) were blue‐sensitive, whereas in *V. variegatus* (485.4 nm), it was green‐sensitive and comparable to *V. moseri* (482.3 nm). Our results indicate that although the four flounder species possess a similar opsin gene repertoire, the SWS2A opsin of the genus *Verasper* is functionally green‐sensitive, while its overall structure remains conserved as a blue‐sensitive opsin. Further, the *rh2‐a* function seems to have been reduced during the evolution of flounders. λ_max_ values of predicted ancestral SWS2A of Pleuronectiformes and Pleuronectidae was 465.4 and 462.4 nm, respectively, indicating that these were blue‐sensitive. Thus, the green‐sensitive SWS2A is estimated to be arisen in ancestral *Verasper* genus. It is suggested that the sensitivity shift of SWS2A from blue to green may have compensated functional reduction in RH2‐A.

## 1 INTRODUCTION

Color vision discriminates spectral differences in photic environments. Among vertebrates, most fishes, amphibians, reptiles, and birds have well‐developed color vision (Bowmaker, 1998; Kawamura, 2011; Yokoyama, 2000). Light colors are perceived by photopigments in cone cells in the retina of the eye (Yokoyama & Starmer, 1996). The ability of cone cells, which receive particular wavelengths of light, depends on the properties of the opsins contained in these cells. Slight differences in opsin structure enable light to be optimally absorbed at different wavelengths. In bony fishes, the opsin repertoire differs among species (Gojobori & Innan, 2009; Yokoyama, 2000). Generally, the surface‐dwelling species, which habit the luminous superficial area of water, have a large cone opsin repertoire, as seen in zebrafish (*Danio rerio*) (Chinen, Hamaoka, Yamada, & Kawamura, 2003), medaka (*Oryzias latipes*) (Matsumoto, Fukamachi, Mitani, & Kawamura, 2006), and guppy (*Poecilia reticulata*) (Kawamura et al., 2016). Conversely, dark deep‐sea fish tend to lose cone cells and corresponding opsins, whereas they possess a high proportion of rod cells, which contribute to dim light vision as in the case of lizardfish (Hope, Partridge, Dulai, & Hunt, 1997).

Some of flounders (flatfishes) change their habitat during ontogeny (Andoh, Watanabe, & Matsubara, 1999). They inhabit the surface layer during larval and juvenile stages, and adults migrate to deep‐water areas. Their color vision system develops in accordance with their metamorphosis and growth. In the winter flounder (*Pseudopleuronectes americanus*), although only green‐sensitive RH2 opsin is expressed in the retina of premetamorphic larva, other opsins, including red‐sensitive LWS, blue‐sensitive SWS2, ultraviolet‐sensitive SWS1, and dim light‐sensitive opsin RH1, are expressed in the retina of postmetamorphic juveniles (Evans, Hárosi, & Fernald, 1993; Hoke, Evans, & Fernald, 2006; Mader & Cameron, 2004).

We recently characterized the visual opsins of the barfin flounder (*Verasper moseri*) inhabiting cold sea basins around Pacific‐facing northeastern Japan (Kasagi et al., 2015). Specifically, fish expressed seven functional opsins—six cone opsins and one rod opsin—in the eyes, and these were categorized into five types of visual opsins, which are found in vertebrates based on homology of amino acid sequence. The wavelengths of the maximum absorption spectra (λ_max_) for each of the reconstituted photopigments were in good agreement with their structural properties. Indeed, RH2‐B and RH2‐C were green‐sensitive opsins (506 and 490 nm, respectively), LWS was a red‐sensitive opsin (552 nm), SWS2B was a blue‐sensitive opsin (416 nm), and SWS1 was an ultraviolet‐sensitive opsin (367 nm).

It is interesting that although SWS2A was a member of the blue‐sensitive opsin according to amino acid sequence, the opsin functioned as a green‐sensitive opsin, because its λ_max_ was 482 nm. This value was close to those of RH2‐B (506 nm) and RH2‐C (490 nm), but far from that of SWS2B (416 nm). The λ_max_ range of SWS2A in other vertebrates falls within 430–460 nm (Matsumoto et al., 2006; Spady et al., 2006). It was therefore conceivable that λ_max_ of SWS2A shifted from the original wavelength to a longer wavelength during the evolution of the barfin flounder (Kasagi et al., 2015). In addition to the seven functional opsins, we found an RH2‐A pseudogene in barfin flounder. The λ_max_ range of the functional RH2‐A in other vertebrates is 450–480 nm (Matsumoto et al., 2006; Spady et al., 2006). Taken together, the pseudogenization of RH2‐A appeared to be related to a functional change caused by a long‐wavelength shift in SWS2A (Kasagi et al., 2015).

Our results suggest that color vision of barfin flounder is well developed to resolve green light, because three of the six cone opsins are green‐sensitive and these three green‐sensitive opsins have different λ_max_—482 nm for SWS2A, 490 nm for RH2‐C, and 506 nm for RH2‐B. This study was conducted to determine whether the green‐sensitive property, including the long‐wavelength shift of SWS2A and pseudogenization of RH2‐A, is specific to barfin flounder or common to all flatfishes. Thus, we investigated the structural and functional properties of opsins in three flatfish species, including the spotted halibut (*Verasper variegatus*, Pleuronectidae), slime flounder (*Microstomus achne*, Pleuronectidae), and Japanese flounder (*Paralichthys olivaceus*, Paralichthyidae), adults of which inhabit water of various depths in the Japanese coastal area (Masuda, Amaoka, Araga, Uyeno, & Yoshino, 1984). Based on the results, impacts of both phylogeny and ecology on the evolution of SWS2A and RH2‐A were evaluated.

## 2 RESULTS

### 2.1 Identification of mRNA and genes for flounder opsins

**Figure 1:**
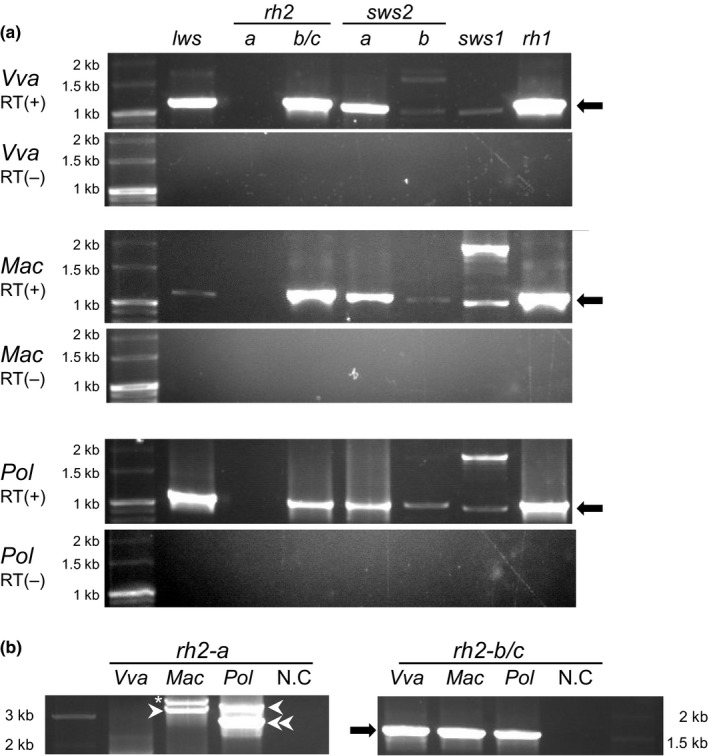
Expression of opsin genes in eyes of adult flounders. (a) RT‐PCR analysis of the opsin genes in ocular RNA from the three flounder species, including *V. variegatus* (Vva), *M. achne* (Mac), and *P. olivaceus* (Pol). Nucleotide sequences for *rh2‐b* and *rh2‐c* were detected in *rh2‐b/c* of Vva and Pol. RT‐PCR amplicons are indicated by arrows. RT (+): RT‐PCR, RT (−): Non‐reverse‐transcribed PCR was the negative control. (b) Genomic PCR analysis of the *rh2‐a* (Mac *rh2‐a* and Pol *rh2‐a2*: white arrowhead, Pol *rh2‐a1*: double arrowhead), *rh2‐b* and *rh2‐c* (arrow) for the flounder genomic DNA. Asterisk is nonspecific amplification. DNA size standards are indicated in kilobases (kb)

Molecular cloning experiments of the eye were conducted to determine whether *V. variegatus*,* M. achne*, or *P. olivaceus* possessed a set of functional opsin genes similar to mRNAs of *V. moseri*. *lws*,* rh2‐b/c*,* sws2a*,* sws2b*,* sws1*, and *rh1* were amplified from total ocular RNA from all three flounder species (Figure 1a). Because *rh2‐b* and *rh2‐c* could not be amplified separately in the present RT‐PCR, these were amplified simultaneously. Two different nucleotide sequences corresponding to *rh2‐b* and *rh2‐c* were detected in the cDNA designated as *rh2‐b/c* amplified from *V. variegatus* and *P. olivaceus*. These results indicated that *rh2‐b* and *rh2‐c* were expressed in the two flounder species. In the case of *M. achne*, nucleotide sequences for *rh2‐b* were detected, but not for *rh2‐c*. The absence of *rh2‐c* was confirmed by Southern hybridization (see next paragraph). No *rh2‐a* mRNA was detected in total ocular RNA of all three flounder species (Figure 1a), whereas DNA fragments containing of a full‐length reading frame for *rh2‐a*, which had no insertion/deletion codon slippage or nonsense mutation in reading frames, including four introns, were amplified from genomic DNA of *M. achne* and *P. olivaceus* (Figure 1b). An additional genomic DNA fragment of *rh2‐a* was also amplified from *P. olivaceus*; therefore, these were distinguished by the terminology *rh2‐a1* and *rh2‐a2* (Figure 1b). However, in *V. variegatus*, a genomic DNA fragment for *rh2‐a* was not detected. Results obtained from genomic PCR and RT‐PCR indicated low or no expression of *rh2‐a* in *P. olivaceus* and *M. achne*, the absence of *rh2‐a* in *V. variegatus*, and the expression of the other opsin genes in these flounder species (Table 1).

**Table 1:** List of flounder visual opsins

Type	Subtype	*V. moseri*	*V. variegatus*	*M. achne*	*P. olivaceus*
		mRNA expression/gene existence			
LWS	*lws*	+	+	+	+
RH2	*rh2‐a*	−/+^a^	−	−/+	−/+
	*rh2‐b*	+	+	+	+
	*rh2‐c*	+	+	−	+
SWS2	*sws2a*	+	+	+	+
	*sws2b*	+	+	+	+
SWS1	*sws1*	+	+	+	+
RH1	*rh1*	+	+	+	+

+: gene exists in genome and mRNA is expressed in eye, −/+: gene exists but mRNA is not expressed or expression is very low, −: gene does not exist.

a *V. moseri rh2‐a* is a pseudogene. Data for *V. moseri* were taken from Kasagi et al. (2015).

**Figure 2:**
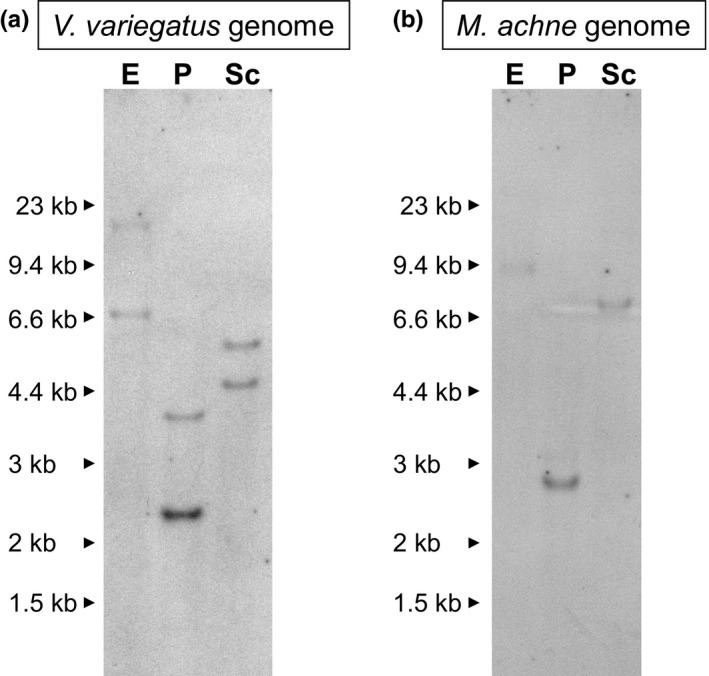
Southern hybridization and analyses of the *V. variegatus* (a) and *M. achne* (b) genomic DNA for *rh2‐b* and *rh2‐c*. *V. moseri rh2‐b* cDNA fragments for exon 1 was used as the probe. Genomic DNA was independently digested with four restriction enzymes, E: *Eco* RI, P: *Pst* I, Sc: *Sac* I. DNA size standards are indicated in kilobases (kb)

The absence of *rh2‐c* in *M. achne* was confirmed with genomic Southern hybridization. *V. variegatus* was used as a reference because this fish expresses both *rh2‐b* and *rh2‐c*. A cDNA fragment of the exon 1 region of *V. moseri rh2‐b* was used for the hybridization probe intended to detect both *rh2‐b* and *rh2‐c* in the flounder species genome, because the probe was able to detect both *V. moseri rh2‐b* and *rh2‐c*, which exhibited 86% similarity (Kasagi et al., 2015). The *V. moseri rh2‐b* probe exhibited 99% and 88% sequence identity to the corresponding regions of *rh2‐b* and *rh2‐c* of *V. variegatus*, and 83% and 95% sequence identity to the corresponding regions of *rh2‐a* and *rh2‐b* of *M. achne*. The stringency of the present hybridization allowed approximately 20% of mismatch (see section 4.4). Southern hybridization of *V. variegatus* genomic DNA detected two bands when DNA was digested with *Eco* RI, *Pst* I, and *Sac* I (Figure 2a). These bands could be referred to as *rh2‐b* and *rh2‐c*, which were amplified by RT‐PCR, and were detected in the hybridization. After hybridization with *V. moseri rh2‐b*, the *V. variegatus* blot was reprobed and then hybridized with the *V. moseri rh2‐a* exon 1 probe (Kasagi et al., 2015); however, no band was observed. It was therefore concluded that *rh2‐b* and *rh2‐c*, but not *rh2‐a*, were present in the *V. variegatus* genome. In the case of *M. achne*, a single band was observed in the digests of the three restriction enzymes (Figure 2b). This band could be referred to as *rh2‐b*, which was also amplified by RT‐PCR. Alternatively, the presence of *rh2‐c* could be excluded. Taking the amplification of *rh2‐a* from genomic PCR into consideration, it was concluded that *rh2‐a* and *rh2‐b*, but not *rh2‐c*, were present in the *M. achne* genome (Table 1).

### 2.2 Characterizations of nucleic acid sequence and amino acid sequence of flounder opsin

**Figure 3:**
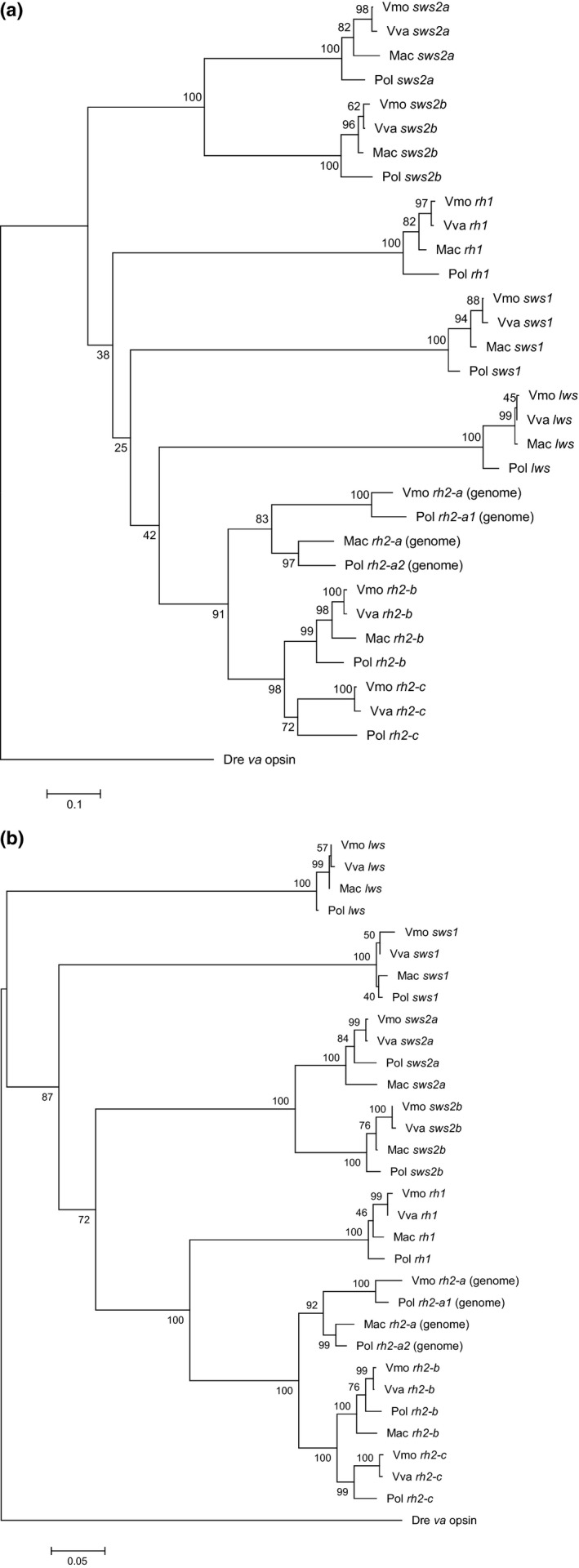
Phylogenetic tree for the barfin flounder opsins (Kasagi et al., 2015) and other flounder opsins (in this study). Trees were constructed by the neighbor‐joining method. (a): calculated from only dS substitution, (b): calculated from only dN substitution. The bootstrap test (500 replicates) scores are shown next to the branches. Vmo: *V. moseri*, Vva: *V. variegatus*, Mac: *M. achne*, Pol: *P. olivaceus*, Dre: *D. rerio*

Phylogenetic trees based on nucleic acid sequence were constructed using the neighbor‐joining method (Figure 3). Two phylogenetic trees using neutral substitution (dS), with dS calculated using synonymous substitution, or non‐neutral substitution (dN), which were calculated from nonsynonymous substitution, were constructed to assess the contribution of dS and dN on the molecular evolution of flounder opsin genes. In the dS tree (Figure 3a), except for *rh2‐a*, the other opsin genes including *sws2a* were localized in a same topology corresponding to the generally accepted phylogeny of flounder species (Nelson, 2006). In the case of *rh2‐a*, two sequence types of *P. olivaceus* (*rh2‐a1* and *rh2‐a2*) were homologous to those of *V. moseri* and *M. achne*, respectively. The topologies of *lws*,* rh2‐a*,* rh2‐c*,* sws2b*, and *rh1* in the dN tree (Figure 3b) were the same as those of the dS tree (Figure 3a), whereas the topologies of *rh2‐b*,* sws1*, and *sws2a* were different from those of the dS tree.

To evaluate selective constraint on the evolution of flounder opsin genes, the branch model comparison of the dN/dS (ω: omega) ratio was conducted using the maximum‐likelihood method (Table 2). When ω was estimated under the hypothesis that ω is constant among branches (1ω model, “model 0” in PAML in Yang, 2007), the ω values for the opsin genes ranged from 0.146 to 0.344. On the other hand, when ω was estimated under the hypothesis that ω is free from branch to branch (free ω model, “model 2” in PAML in Yang, 2007), the ω values for the opsin genes ranged from 0.075 to 1.19, except when there was no synonymous substitution (ω = 999) or nonsynonymous substitution (ω = 0.0001) in a branch. Significant differences in the likelihood score for each gene between the 1ω model and the free ω model were observed for *sws2a* and *sws1* by χ^2^ test. These results suggested that *sws2a* and *sws1* among the eight opsin genes of flounders were biased from their likely phylogeny.

**Table 2:** Results of tests for the branch model comparison of flounder visual opsins

Gene	1ω model		Free ω model	Likelihood ratio test		
	ω	InL	ω_1_, ω_2_, … ω_5_	InL	ΔInL	*p* (χ^2^ test)
*lws*	0.146	−1654.4	0.0001, 999, 0.0001, 999, 0.130	−1651.2	6.489	.166
*rh2‐a*	0.186	−2347.6	0.172, 0.299, 0.21, 0.151, 0.144	−2346.3	2.547	.636
*rh2‐b*	0.250	−1846.4	0.199, 0.311, 0.0001, 0.406, 0.174	−1844.5	3.789	.435
*rh2‐c*	0.147	−1814.5	0.809, 0.182, 0.136	−1813.6	1.674	.433
*sws2a*	0.344	−1951.7	0.309, 999, 0.0001, 0.659, 0.194	−1946.7	9.963	.041*
*sws2b*	0.248	−1786.5	0.443, 0.0001, 0.160, 0.787, 0.173	−1783.6	5.734	.220
*sws1*	0.215	−1657.5	0.343, 1.190, 0.0001, 0.257, 0.075	−1651.8	11.304	.023*
*rh1*	0.240	−1836.8	0.400, 0.278, 0.0001, 0.505, 0.167	−1833.4	6.754	.150

ω1, ω2… ω5 correspond to w for each branch (see section 4.7). ω = 0.0001 means no dN in branch, ω = 999 means no dS in branch.

### 2.3 Functional analysis of flounder SWS2A by in vitro photopigment reconstitution

**Figure 4:**
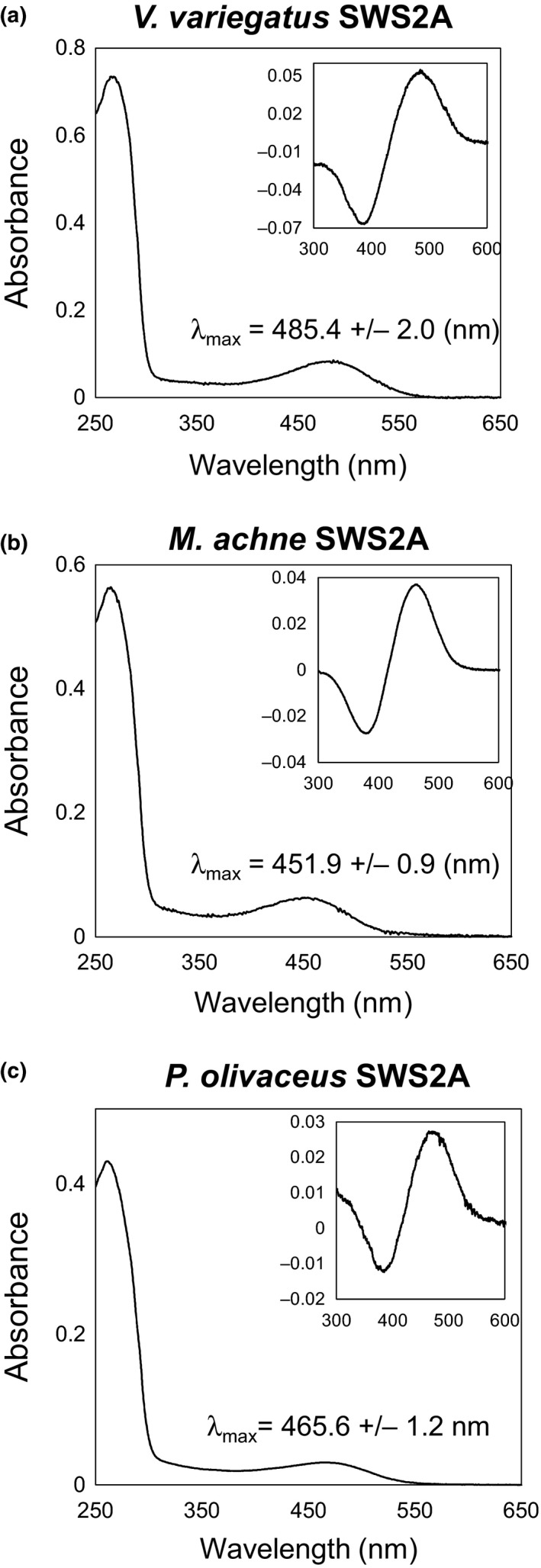
Absorption spectra of the reconstituted opsin photopigments of flounder SWS2A measured in the dark. (a): *V. variegatus*, (b): *M. achne*, and (c): *P. olivaceus*. λ_max_ values are shown with standard deviations. Insets are dark–light difference spectra

To assess functional properties of light absorption, λ_max_ values of flounder SWS2A were determined by *in vitro* reconstitution of visual pigments and compared with *V. moseri* (Vmo) SWS2A. As a result, λ_max_ values of each SWS2A were 485.4 nm for *V. variegatus* (Vva) SWS2A, 451.9 nm for *M. achne* (Mac) SWS2A, and 465.6 nm for *P. olivaceus* (Pol) SWS2A (Figure 4). Compared to Vmo SWS2A (λ_max_: 482.3 nm), the λ_max_ value of Vva SWS2A was slightly longer and categorized as a green‐sensitive opsin, and those of Mac SWS2A and Pol SWS2A were shorter and categorized as a blue‐sensitive opsin. These results suggested that SWS2A of genus *Verasper* has functionally shifted from the ordinary blue‐sensitive opsin.

**Figure 5:**
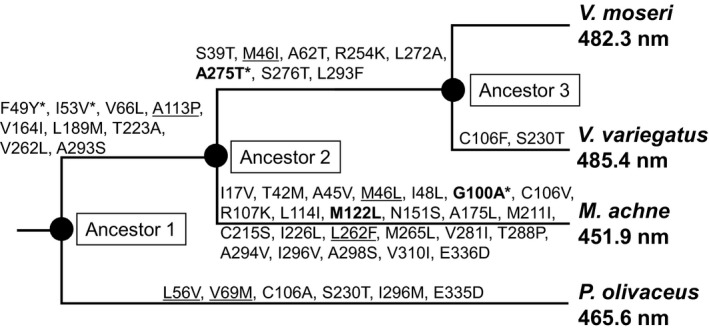
Amino acid replacements in the SWS2A pigments inferred by the JTT model. The λ_max_ values are referred from Kasagi et al. (2015) and this study (Figure 4). The amino acid replacements that have inference probabilities lower than 0.9 are underlined; replacements that occurred in known tuning sites (Table 3) are shown in bold; replacements that occurred in retinal surrounding sites (Palczewski et al., 2000) are asterisked. The phylogeny of the four flounders was depicted on the basis of Nelson (2006)

To investigate the evolutionary background of *Verasper* SWS2A from blue‐sensitive to green‐sensitive opsin, ancestral amino acid sequences were inferred using the maximum‐likelihood method with the sequences of four flounder SWS2As (Figure 5). The number of amino acid replacements from ancestor 1 (ancestral SWS2A of Pleuronectiformes) to ancestor 2 (ancestral SWS2A of Pleuronectidae) and Pol SWS2A, from ancestor 2 to ancestor 3 (ancestral SWS2A of genus *Verasper*) and Mac SWS2A, and from ancestor 3 to Vmo SWS2A and Vva SWS2A was inferred as shown in Figure 5. Ancestor 3 was inferred to have the same amino acid sequence as Vmo SWS2A; therefore, the λ_max_ value of ancestor 3 would be 482.3 nm.

**Figure 6:**
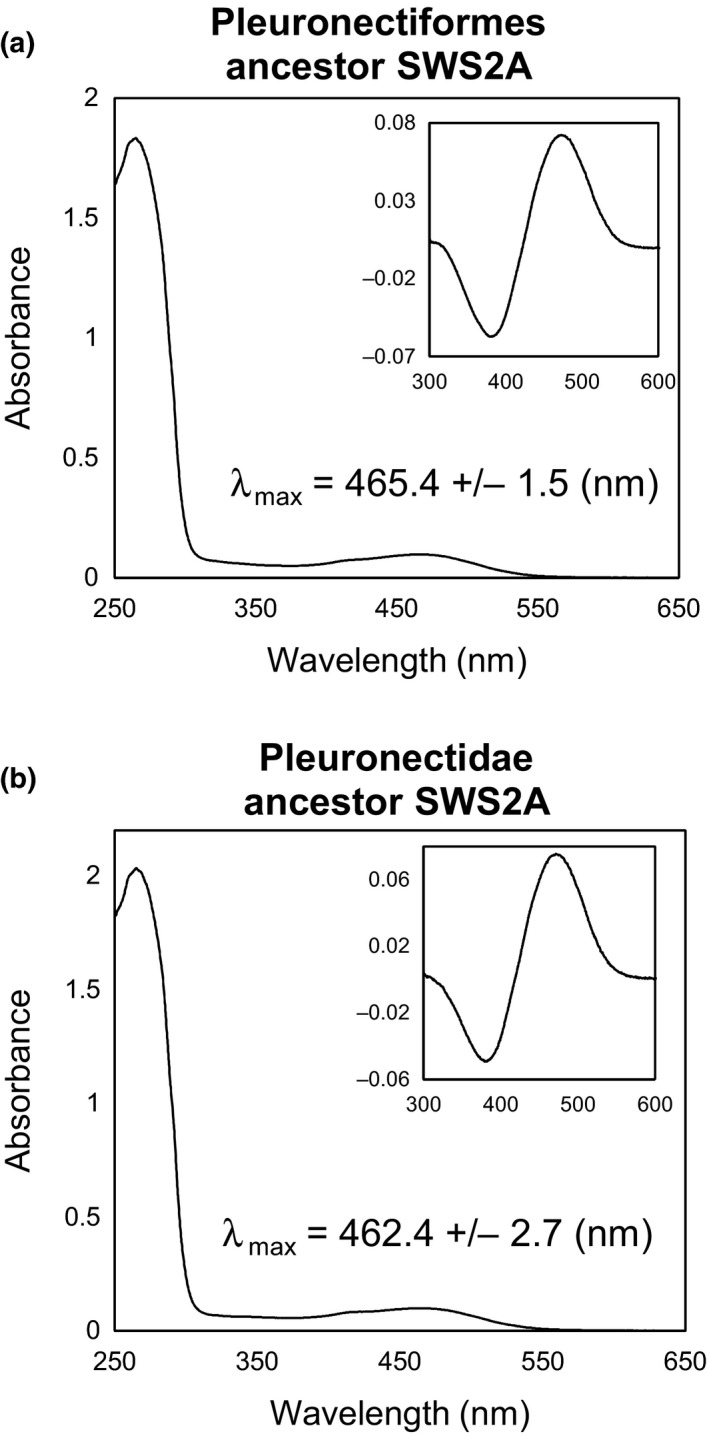
Absorption spectra of the reconstituted opsin photopigments of ancestral flounder SWS2A measured in the dark. (a): Pleuronectiformes ancestor (ancestor 1 in Figure 5), (b): Pleuronectidae ancestor (ancestor 2 in Figure 5). λ_max_ values are shown with standard deviations. Insets are dark–light difference spectra

To estimate λ_max_ values of ancestors 1 and 2, cDNA for ancestor 1 containing six nonsynonymous mutations and that of ancestor 2 containing eight nonsynonymous mutations were prepared from Pol SWS2A and Vmo SWS2A, respectively, by PCR‐based site‐directed mutagenesis and encoded ancestral SWS2As were expressed for *in vitro* reconstitution. As a result, λ_max_ values of ancestors 1 and 2 were 465.4 and 462.4 nm, respectively (Figure 6), suggesting that these ancestors were categorized as being blue‐sensitive opsin.

**Figure 7:**
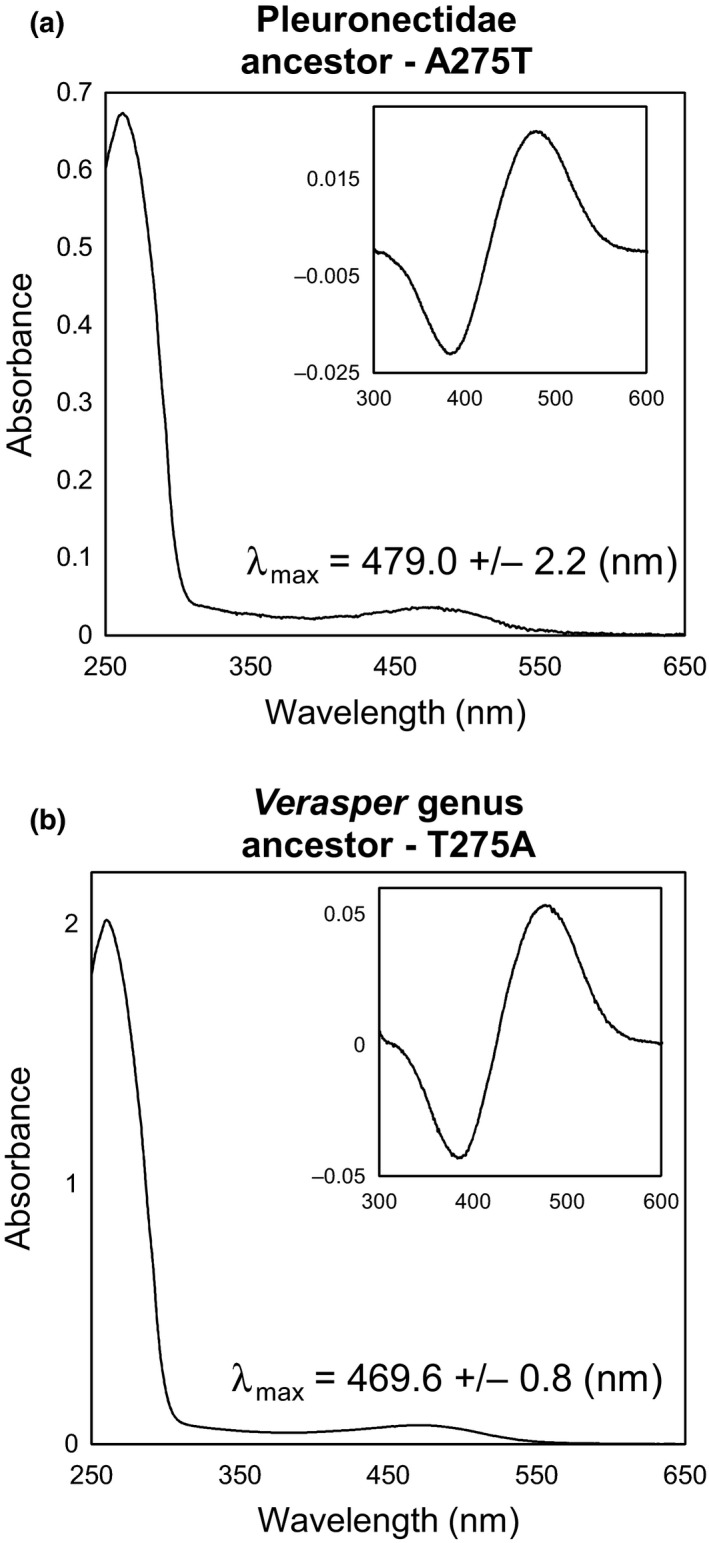
Absorption spectra of the mutant forms of the Pleuronectidae ancestor SWS2A (a) and the *Verasper* genus ancestor (b). λ_max_ values are shown with standard deviations. Insets are dark–light difference spectra

To survey amino acid residues causing the shift of SWS2A from blue sensitive to green sensitive, amino acid substitution Ala or Thr at position 275 was investigated, because this site is known to act as a spectral tuning site (Yokoyama & Tada, 2003; Yokoyama et al., 2007) and a retinal surrounding site (Palczewski, Kumasaka, Hori, & Behnke, 2000). In addition, the 275Thr codon (ACC) was common between *V. moseri* and *V. variegatus*, suggesting the nucleotide substitution was a single event in the *Verasper* genus ancestor. The A275T mutation lengthened the λ_max_ 462.4 nm of ancestor 2–479 nm, and T275A shortened the λ_max_ value 482.3 nm of ancestor 3 to 469.6 nm (Table 4, Figure 7). The results suggested that the occurrence of Thr275 was critical to the shifting λ_max_ value of SWS2A to a longer wavelength.

## 3 DISCUSSION

### 3.1 Characteristics of flounder visual opsins

We identified the genomic repertoire of visual opsin for the three flounder species, including *V. variegatus*,* M. achne*, and *P. olivaceus*. The present results, together with our previous results for *V. moseri*, provide at least five properties of flounder opsins. (1) All four species possess *lws*,* rh2‐b*,* sws2a*, s*ws2b*,* sws1*, and *rh1* opsin in their genome and corresponding opsin mRNA are expressed in eyes. (2) Although *V. variegatus*,* V. moseri*, and *P. olivaceus* possess *rh2‐c*,* M. achne* lacks the gene. (3) *V. moseri*,* M. achne*, and *P. olivaceus* possess *rh2‐a*, but mRNA expression in these fishes was very low or not detected. (4) *V. variegatus* lacks *rh2‐a*. (5) *rh2‐a* is located in two loci, whereas both of them are not expressed in the eyes of adult specimens of *P. olivaceus*. These four lines of evidence suggest that biological significance of *rh2‐a* is relatively low in flounders and those of *rh2‐c* are incidentally low in *M. achne*, whereas the other opsin genes are functionally expressed.

Upon comparing amino acid sequences among four flounders, there were amino acid differences for known spectral tuning sites only in the SWS2A opsin. Therefore, spectral sensitivity of SWS2A is hypothesized to be different among the four species. This hypothesis was validated by *in vitro* reconstitution; *V. moseri* SWS2A and *V. variegatus* SWS2A are green‐sensitive, whereas *M. achne* SWS2A and *P. olivaceus* SWSA are blue‐sensitive. Furthermore, we deduced that the key amino acid substitution for the long‐wavelength sensitivity shift was introduced in the lineage of a common ancestor in the genus *Verasper*.

### 3.2 Evolution of RH2 subtypes

**Figure 8:**
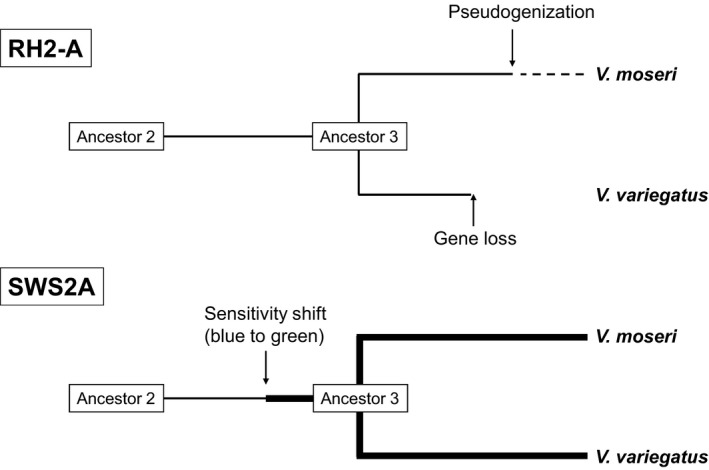
Phylogenetic schematics of RH2‐A and SWS2A in the genus *Verasper*. Ancestor 2 is a Pleuronectidae ancestor, and ancestor 3 is a genus *Verasper* ancestor

No detection of the mRNA for *rh2‐a* of *P. olivaceus* and *M. achne rh2‐c* appeared to be caused by low expression levels of these genes, because no nonsense mutation was observed in their coding frame. In the case of *V. moseri*,* rh2‐a* was a pseudogene with a single nonsense mutation, in which neither insertion nor deletion of nucleotides was observed. These structural properties suggest that the pseudogenization was occurred in an ancestral strain of *V. moseri* (Figure 8). In spite of the occurrence of the stop codon in the coding frame of *rh2‐a*, overall amino acid sequences of the *V. moseri* RH2‐A were well conserved (Kasagi et al., 2015). It was therefore concluded that the nonsense mutation occurred after the divergence of *V. moseri* from Ancestor 3 (Figure 8). In contrast to *V. moseri*, neither PCR nor Southern hybridization provided *rh2‐a*‐like sequences in genomic DNA of *V. variegatus*. Because of the presence of *rh2‐a* in *M. achne*, as well as *V. moseri*, the absence of the gene in *V. variegatus* is supposed to have occurred after diversification from ancestor 3 and that gene was deleted perhaps by unequal crossing over. Consequently, pseudogenization of *rh2‐a* in *V. moseri* and gene loss in *V. variegatus* are thought to be independent events in each species (Figure 8). Taken together, none or very low *rh2‐a* expression in the eyes is considered a common characteristics of these four flounder species, suggesting that functional significance of *rh2‐a* was decreased during the course of Pleuronectiformes evolution.

*rh2‐c* was detected in *V. moseri*,* V. variegatus*, and *P. olivaceus*, but not in *M. achne*. Considering phylogenetic relationships of flounders used in this study, *rh2‐c* may have been secondarily lost in *M. achne* after diversification from a common ancestor shared with genus *Verasper*. Among three subtypes of *rh2*, only *rh2‐b* is consistently present as a functional gene throughout the four flounder species, suggesting that the presence of the three functional subtypes was not always necessary for color vision in the Pleuronectiformes.

### 3.3 Evolution of SWS2A

Although SWS2A is categorized into blue‐sensitive opsin based on amino acid sequence comparison as shown by λ_max_ in *M. achne* (451.9 nm), *P. olivaceus* (465.6 nm), and other teleosts (430–460 nm), those from genus *Verasper* act as green‐sensitive opsin because λ_max_ values of SWS2A for *V. moseri* and *V. variegatus* were 482 and 485 nm, respectively. This suggests that the shift in λ_max_ from blue sensitive to green sensitive may have occurred in a common ancestor of the genus *Verasper* (Figure 8). Regarding site‐directed mutagenesis based on the putative evolution from an ancestor of Pleuronectidae (ancestor 2) to an ancestor of the genus *Verasper* (ancestor 3), reconstitution experiments revealed that a single mutation from Ala to Thr at position 275 significantly contributed to the green shift. The importance of the replacement could also be explained by an amino acid sequence comparison for SWS2A among the four flounder. Specifically, at one of the tuning sites—position 275, SWS2A of *M. achne* and *P. olivaceus* containing Ala is blue sensitive, whereas that of *V. moseri* and *V. variegatus* containing Thr is green‐sensitive (Table 3). The other replacements, which were simultaneously introduced to a single mutant cDNA, also contributed to the green shift, whereas their total effects were lower than the single mutation at 275 (Table 4). Thus, among several representative tuning sites shown in Table 3, this study demonstrated the predominant contribution of position 275 in the green shift of SWS2A in the genus *Verasper*.

**Table 3:** Representative amino acids sites involved in the spectral sensitivity of acanthopterygian SWS2A opsins

Tuning site		*V. moseri*	*V. variegatus*	*M. achne*	*P. olivaceus*
	λ_max_ (nm)	482.3	485.4	451.9	465.6
52		F	F	F	F
55		V	V	V	V
58		T	T	T	T
99		V	V	V	V
100		**G**	**G**	**A**	**G**
122		**M**	**M**	**L**	**M**
124		T	T	T	T
170		A	A	A	A
213		L	L	L	L
275		**T**	**T**	**A**	**A**
301		S	S	S	S

A list of amino acid compositions of tuning sites are taken from Nakamura et al. (2013). Number of amino acid residues was taken from flounder SWS2A. Boldface letters indicate amino acid residues that are inconsistent among the four flounder species.

**Table 4:** Effects of amino acids substitutions in ancestral flounder SWS2A

	Substitution	λ_max_ (nm)	Δ λ_max_ from Pleuronectidae ancestor (nm)
Pleuronectidae ancestor	–	462.4	–
	A275T	479.0	+16.6
	S39T, M46I, A62T, R254K, L272A, S276T, L293F	469.6	+7.2
Genus *Verasper* ancestor	–	482.3	+19.9

Mutation of SWS2A in *V. moseri* and *V. variegatus* leading this opsin to be green sensitive may have occurred in their common ancestor, because SWS2A from the two species shares similar properties in light absorption and amino acid sequences, especially at representative tuning sites. These properties appear to have emerged in a common ancestor in the genus *Verasper* during evolution from ancestor 2 to ancestor 3. The branch model comparison indicated that flounder SWS2A was under selective constraints throughout the flounder lineage from the viewpoint of molecular evolution. This analysis again proposed the importance of the mutation of A275T at a tuning site, which may have occurred as a chance event.

### 3.4 Relationship between ecology and vision in flounders

In teleosts, RH2 and SWS2 opsins show increased divergence in structure and function compared to SWS1 and LWS opsins, by copy number variation in each gene and genetic distance between orthologues (Gojobori & Innan, 2009). These variations are estimated to be caused by different selective pressure among opsins; LWS and SWS1, which have a conservative role to detect long and short wavelengths, respectively, have been under high selective pressure not to change the visible wavelength range. In contrast, RH2 and SWS2 are estimated to be under relatively low selective pressure and have evolved to perform a variety of functions, which likely have contributed to their ecological adaptation by shifting their sensitivity and/or changing their copy numbers.

The occurrence of three green‐sensitive opsins in the genus *Verasper* may be related to their ecological properties. In general, adult flounders migrate from the deeper sea toward the shore, where they spawn. After hatching, larval flounders with symmetrical shape spend their planktonic stage close to the shore. After metamorphosis into asymmetrical shaped juveniles, the flounders move to the bottom of the shallow waters, where they begin the bottom‐dwelling life. As they continue growing, they migrate to the deeper sea, where sunlight is reduced, depending on the depth and seawater composition (Minami & Tanaka, 1992; Münzing, 1968; Wada, Mitsunaga, Suzuki, Yamashiya, & Tanaka, 2006). In a clear and low‐organic‐matter ocean, the reduction in the intensity of sunlight occurs in longer‐wavelength light because of absorption, and the shorter‐wavelength light also decreases because of Rayleigh scatter. Thus, the deeper the flounders live in the ocean, the more predominantly the middle‐wavelength light they receive (Levine and MacNichol, 1982). Therefore, it is assumed that the richness of green‐sensitive opsins in the genus *Verasper* may be related to green light predominance in the deeper sea. Among the three genera of flounders examined, the number of green‐sensitive opsins may have increased by chance in *Verasper* during evolution, compared to *Microstomus* and *Paralichthys*. At least, it is assumed that the sensitivity shift of SWS2A from blue to green may have compensated the functional reduction in RH2‐A.

## 4 MATERIALS AND METHODS

### 4.1 Fishes and preparation of tissue samples

Adult spotted halibut, *V. variegatus*, and slime flounder, *M. achne*, were caught in Sendai Gulf and kept in Tohoku National Fisheries Research Institute, Japan Fisheries Research and Education Agency, Miyagi, Japan. Adult Japanese flounder, *P. olivaceus,* were bred at the Kanagawa Prefectural Fisheries Technology Center, Kanagawa, Japan. The average body weight of spotted halibut, slime flounder, and Japanese flounder was 382.8, 175.0, and 144.5 g, respectively. Fish were treated according to Guidelines for the Care and Use of Animals of the Kitasato University. Prior to tissue collection, all individuals were anesthetized by 0.05% 2‐phenoxyethanol (Wako, Osaka, Japan). The eyes and muscle tissues were immediately dissected and frozen on dry ice; the samples were stored at −80°C until analysis.

### 4.2 Nucleic acid preparation and amplification

Eyes were homogenized using MixerMill MM300 (Reche, Haan, Germany), and total RNA was isolated using ISOGEN2 reagent (Wako). Total RNA was treated with RNase‐free DNase (TaKaRa, Otsu, Japan) to eliminate genomic DNA contamination and then purified by phenol–chloroform extraction, followed by isopropanol precipitation. First‐strand cDNA was prepared using the PrimeScript II 1st Strand Synthesis kit (TaKaRa). Genomic DNA was isolated from the muscle tissue according to a previously described method (Sambrook & Russell, 2001). Polymerase chain reaction (PCR) to amplify cDNA and genomic DNA was performed using the TaKaRa Ex Taq Hot Start Version kit (TaKaRa) under conventional conditions. Amplified DNA fragments were purified using the Nucleospin gel and PCR cleanup kit (Macherey‐Nagel, Düren, Germany) after agarose gel electrophoresis. Custom oligonucleotides designed for PCR (Table S1) were synthesized at Life Technologies (Carlsbad, CA, USA). The full‐length cDNA fragments were subcloned into pGEM‐T Easy vectors (Promega, Fitchburg, WI, USA) followed by standard alkaline‐SDS plasmid preparation (Sambrook & Russell, 2001).

### 4.3 cDNA, genomic DNA cloning of opsins

For all opsin genes except *rh2‐a*, the opsin cDNAs containing the entire coding region were amplified from the first‐strand cDNA that was synthesized from the eye RNA. Primers were designed based on the nucleotide sequences of opsin genes of the barfin flounder (GenBank accession numbers, LWS: AB930175, RH2‐B: AB930177, and RH2‐C: AB930178, SWS1: AB930179, SWS2A: AB930180, SWS2B: AB930181, RH1: 930176). In the case of Mac *rh2‐a*, a partial DNA segment was amplified from genomic DNA by PCR using “internal forward” and “internal reverse” primers designed from the barfin flounder *rh2‐a* (Table S1). Subsequently, upstream and downstream DNA fragments were amplified by inverse PCR (see Sambrook & Russell, 2001 for the principles). Finally, the entire sequence was amplified from genomic DNA. The full‐length cDNAs and *rh2‐a* genomic DNA were cloned into the pGEM‐T vector. DNA sequencing analyses were conducted for the purified PCR products and the cloned DNA using a Big Dye Terminator v3.1 cycle sequencing kit (Applied Biosystems, Foster City, CA, USA) and ABI 3130 xl genetic analyzer (Applied Biosystems).

### 4.4 Genomic southern hybridization

Approximately 10 μg of genomic DNA per lane was digested with the restriction enzymes *Eco* RI, *Pst* I, or *Sac* I (TaKaRa) and electrophoresed on a 0.7% agarose gel. DNA samples were then transferred to a Hybond‐N+ nylon membrane (GE Healthcare, Buckinghamshire, UK) using a Model 785 vacuum blotter (Bio‐Rad, Hercules, CA, USA). DNA was fixed to the membrane using an XL‐1500 UV cross‐linker (Spectroline, NY, USA). As a hybridization probe, exon 1 of the barfin flounder *rh2‐b* gene was used (previously described in Kasagi et al., 2015). Amplified cDNA was labeled with an Alkphos‐Direct labeling kit (GE Healthcare), and hybridization was performed according to the manufacturer's instructions. Stringent washing was conducted at 60°C in primary buffer wash (per the manufacturer's instructions), which contained 0.2 M of NaCl and 0.1% of SDS, which allowed approximately 20% mismatch (Sambrook & Russell, 2001). Signals were detected using CDP‐Star (GE Healthcare) and Hyperfilm ECL (GE Healthcare).

### 4.5 Phylogenetic tree construction

Phylogenetic relationships among the nucleotide sequences were inferred for the barfin flounder (Kasagi et al., 2015), spotted halibut (*lws*: LC209595, *rh2‐b*: LC209601, *rh2‐c*: LC209604, *sws2a*: LC209609, *sws2b*: LC209612, *sws1*: LC209598, *rh1*: LC209606), slime flounder (*lws*: LC209596, *rh2‐a*: LC209814, *rh2‐b*: LC209602, *sws2a*: LC209610, *sws2b*: LC209613, *sws1*: LC209599, *rh1*: LC209607), and Japanese flounder (*lws*: LC209597, *rh2‐a1*: LC209812, *rh2‐a2*: LC209813, *rh2‐b*: LC209603, *rh2‐c*: LC209605, *sws2a*: LC209611, *sws2b*: LC209614, *sws1*: LC209600, *rh1*: LC209608) opsins and zebrafish VA opsin as an outgroup (NM_131586), using MEGA 5.1 software (Tamura et al., 2011). The evolutionary distance of nucleic acid synonymous–nonsynonymous substitutions was estimated using the Nei‐Gojobori method (Nei & Gojobori, 1986), and a phylogenetic tree was constructed by applying the neighbor‐joining method (Saitou & Nei, 1987). The reliability of tree topology was evaluated by bootstrap analysis with 500 replications.

### 4.6 Amino acid comparison of flounder opsin cDNAs

Representative amino acid sites involved in the light sensitivity of LWS, SWS1, SWS2, RH1, and RH2 opsins were compared among the four flounder species: *V. moseri*,* V. variegatus*,* M. achne*, and *P. olivaceus*. Tuning sites were taken from a previous study (Nakamura et al., 2013). The results for SWS2A opsin are shown in Table 3, and the others were omitted because there were no differences among flounder species.

### 4.7 Branch model dN/dS comparison of opsin by maximum‐likelihood method

The dN/dS ratio (ω) is an indicator for evaluating selection pressure. The ω values of the flounder opsin genes were estimated by the branch model with the CODEML program in PAML version 4.8 (Yang, 2007) with PAMLX version 1.3.1 (Xu & Yang, 2013). Alignments of opsin cDNA nucleotide sequences were constructed as stated in section 4.1. The analysis was conducted based on the tree topology of the morphological classification (Nelson, 2006): ([*V. moseri*,* V. variegatus*], *M. achne*,* P. olivaceus*). In the case of *rh2‐a*, tree topology was set as following neighbor‐joining tree topology in Figure 3 ([*V. moseri*,* P. olivaceus* 1], [*M. achne*,* P. olivaceus* 2]). In the case of *rh2‐c*, tree topology was set as (*V. moseri*,* V. variegatus*,* P. olivaceus*) because *M. achne rh2‐c* was absent.

We constructed two hypotheses for the evaluation of the omega score of each opsin gene: (1) The ω value was uniform among every branch (1 ω model), and (2) the omega values varied among branches (free ω model). When likelihood scores between the two models were compared, if the likelihood score of the multi‐omega model was higher than that of the single omega model, then the opsin gene had evolved in an unbalanced (non‐neutral) manner (Wang et al., 2011). To assess the significance between the two models, log‐likelihood ratio tests for each opsin genes were conducted by χ^2^ test to the 2* (delta likelihood ratio), with the significance level set at 5%. Parameter number for model 1 calculation was 7 and was 11 for model 2; thus, the degrees of freedom was four (in the case of RH2‐C, parameters for models 1 and 2 were five and seven respectively, so the degrees of freedom was two).

### 4.8 Inference of ancestral opsin sequence

Ancestral states of SWS2A opsin were inferred using the maximum‐likelihood method (Nei & Kumar, 2000) under the JTT matrix‐based model (Jones, Taylor, & Thornton, 1992). In addition to the barfin flounder, spotted halibut, slime flounder, and Japanese flounder, the following acanthopterygian species SWS2A amino acid sequences were used as outgroups; Nile tilapia (*Oreochromis niloticus*) *sws2a* (GenBank Accession No.: JF262088), Cichlid (*Maylandia zebra*) *sws2a* (ADW80520), bluefin killifish (*Lucania goodei*) *sws2a* (AY296737), and medaka (*Oryzias latipes*) *sws2a* (AB223056). The set of possible amino acids states at each ancestral nodes were inferred based on their inferred likelihood. The initial tree was inferred from morphological phylogeny (Nelson, 2006) and molecular phylogeny (Betancur & Orti, 2014). The rates among sites were treated as uniform among sites. The analysis involved eight amino acid sequences. Evolutionary analyses were conducted in MEGA5 (Tamura et al., 2011).

### 4.9 Reconstitution of opsin photopigment

The entire coding regions of flounder *sws2a* opsin cDNAs were amplified from the pGEM‐T clones by PCR using the primer pairs listed in Table S1. Subsequent procedures such as construction of expression vectors, expression of the vector in the COS‐1 cell, and photopigment reconstitution were performed as described previously in Kasagi et al. (2015).

### 4.10 Site‐directed mutagenesis

The cDNA clones of the barfin flounder, slime flounder, and Japanese flounder *sws2a* in pMT5 expression vector (section 4.9) were used as template DNA for site‐directed mutagenesis. Primers used for mutagenesis are listed in Table S2. Mutagenesis reactions were carried out using TaKaRa PrimeSTAR Max DNA Polymerase (TaKaRa) with the following thermal cycles: 95°C for 10 s, 25 cycles of 95°C for 10 s, 59°C for 10 s, 72°C for 1 min. After the thermal reaction, the product was digested with *Dpn* I restriction enzyme (TaKaRa), purified by agarose gel extraction (see 4.2), and transformed into *E. coli*. Mutated DNAs were sequenced after plasmid DNA preparation, to confirm that no unexpected mutations were incorporated.

## DATA AVAILABILITY

Nucleic acid sequence data which obtained in this study are available on GenBank (accession numbers: LC209595‐LC209614, LC209812‐LC209814). Detailed information is described in section 4.5.

## CONFLICT OF INTEREST

None declared.

## AUTHOR CONTRIBUTIONS

AT provided the general design of the study. SK and KM collected samples. Molecular cloning of opsin genes, phylogenetic analyses, *in vitro* reconstitutions, and manuscript drafting were carried out by SK, KM, and AT participated in manuscript revising.
