# Supplementary material for: Green‐shifting of SWS2A opsin sensitivity and loss of function of RH2‐A opsin in flounders, genus Verasper
Source: Ecol Evol. 2017 Dec 27;8(2):1399–410. doi: 10.1002/ece3.3745 (PMC5773313; doi:10.1002/ece3.3745)
Supplement: Supplementary file 1 [file ECE3-8-1399-s001.pdf]

Table S1. List of oligonucleotide PCR primers used for cDNA cloning

Gene		Nucleotide sequences
lws	PCR-forward	ACTGTTGAGGGCTCTCCTGC
	PCR-reverse	CCCCCCAAATTAGAGAACATGGAG
rh2-a	PCR-forward	CAATTCAATCCTAACTAAGCAAAG
	PCR-reverse	TGTTGGGAATGGTCTGTGT
	Internal-forward	CTGGCTTCCCCATCAACGCTCT
	Internal-reverse	TGGTCAGCATGCAGTTACGGAACT
	Inverse-reverse	AGGAGGAATAGCGCAGGAGG
	Inverse-forward	GTCATTCGTCTTGTTCATGTTCACCTG
	Mac-full-forward	ATGGAGAACGGCACAGAGGGCCAGAA
	Mac-full-reverse	TTAGGAAACAGAGGACACTTCTGTCTTGCTGG
rh2-b/c	PCR-forward	ATGTCTTGGGACGGAGGAATCGA
	PCR-reverse	ACACAGAGGACACCTCTGTCT
	5’RACE-Rev	AGAATCTTGTACATGATGTGATCCA
	3’RACE-For	GTACCATATGCCACTTTCGCCGC
	Pol-full-for	GCTCCAGCGAAGGTGAACGGA
	Pol-full-rev	GAGACAGTGGAAAGGAAAATGTCCAGG
sws2a	PCR-forward	CTTGTTTGCCCGTGGGGGAAA
	PCR-reverse	CTGTGGAAAACGTTGTGGATGTTCAG
	pMT-forward	GTACGA*GAATTCC*ACCATGAAGCACGGCCGGGT
	pMT-reverse	TGGTTC*GTCGAC*GCAGGCCCAACTTTGGAGACTTCGG
sws2b	PCR-forward	CATTTGACAAACAACCAGAGG
	PCR-reverse	CTTTCAGTGCTATTTACACTCTATG
sws1	PCR-forward	CATGGGGAGAAAGCTCAGGTTC
	PCR-reverse	CATCCGACATCACTTCTAATTTGTTCAAACA
rh1	PCR-forward	CGCAAACCGCAAGCCGCAA
	PCR-reverse	CATGGAGCCTTTGTAAATGGCCCT

All nucleotides sequences are denoted in 5’-3’ direction. For pMT primer, *Eco*R I or *Sal* I sites are in italic, start codon is underlined.

Table S2. List of oligonucleotide PCR primers used for site-directed mutagenesis

To generate	substitution		Nucleotide sequences
Pleuronectidae ancestor	39S	Sense	CCATCTAGCAAGCTCAGGCACCT
		Antisense	AGGTGCCTGAGCTTGCTAGATGG
	46M	Sense	CACCTTCTACGCCATGGCCATATACATGT
		Antisense	ACATGTATATGGCCATGGCGTAGAAGGTG
	62A	Sense	ACTAGCATCAATGCACTCACCATCCTG
		Antisense	CAGGATGGTGAGTGCATTGATGCTAGT
	254R	Sense	GAAGGCAGAGAGGGAGGTGACCAG
		Antisense	CTGGTCACCTCCCTCTCTGCCTTC
	272L, 275A, 276S	Sense	TGCTGGTTGCCCTACGCTTCCTTTGCC
		Antisense	GGCAAAGGAAGCGTAGGGCAACCAGCA
	293L	Sense	TTCGACCTGAGATTGGCTACTATCCCA
		Antisense	TGGGATAGTAGCCAATCTCAGGTCGAA
Pleuronectiformes ancestor	56V	Sense	TTTTTTATATTTGTTTTGGGCACTAGCATCAAC
		Antisense	GTTGATGCTAGTGCCCAAAACAAATATAAAAAA
	69V	Sense	ATCGTGTGCACCGTGAAATACAAGAAG
		Antisense	CTTCTTGTATTTCACGGTGCACACGAT
	106C	Sense	TGCTGCGCATTTTCATGCAGATATTTTATC
		Antisense	GATAAAATATCTGCATGAAAATGCGCAGCA
	230S	Sense	ATCTTCTGCTACTCACAGCTGCTCATC
		Antisense	GATGAGCAGCTGTGAGTAGCAGAAGAT
	296I	Sense	AGATTGGCTACTATCCCAGCTGTCTTC
		Antisense	GAAGACAGCTGGGATAGTAGCCAATCT
	335E	Sense	GGTGGAGGTGATGATGAAGAATCTTCAACA
		Antisense	TGTTGAAGATTCTTCATCATCACCTCCACC
Pleuronectidae ancestor to *Verasper* genus ancestor	275T	Sense	TGGTTGCCCTACACTTCCTTTGCCCTT
		Antisense	AAGGGCAAAGGAAGTGTAGGGCAACCA
	275A	Sense	TGGGCGCCCTACGCTACCTTTGCCCTT
		Antisense	AAGGGCAAAGGTAGCGTAGGGCGCCCA

All nucleotides sequences are denoted in 5’-3’ direction. Designated mutations (codons) are underlined.
